# Metal Permeation into Multi-layered Graphene Oxide

**DOI:** 10.1038/srep03647

**Published:** 2014-01-13

**Authors:** Chikako Ogata, Michio Koinuma, Kazuto Hatakeyama, Hikaru Tateishi, Mohamad Zainul Asrori, Takaaki Taniguchi, Asami Funatsu, Yasumichi Matsumoto

**Affiliations:** 1 Graduate School of Science and Technology, Kumamoto University, 2-39-1 Kurokami, Kumamoto, 860-8555, Japan; 2 JST, CREST, K's Gobancho 6F, 7 Gobancho, Chiyoda-ku, Tokyo, 102-0076, Japan; 3 Materials Science Groups and Laboratory, Deparment of Physics, Sepuluh Nopember Institute of Technology, Surabaya 60111, Indonesia

## Abstract

Understanding the chemical and physical properties of metal/graphene oxide (M/GO) interfaces is important when GO is used in electronic and electrochemical devices because the metal layer must be firmly attached to GO. Here, permeation of metal from the surface into GO paper bulk at the M/GO interface was observed at room temperature for metals such as Cu, Ag, Ni, Au, and Pt. Cu, Ag, and Ni quickly permeated GO as ions into the bulk under humid conditions. At first, these metals changed to hydrated ions as a result of redox reactions (with reduction of GO) at the surface, and then permeated the interlayers. Au and Pt were observed to permeate GO as atoms into the GO bulk at room temperature, although the permeation rates were low. These surprising results are considered to be due to the presence of many defects and/or edges with oxygenated groups in the GO paper.

Strong adhesion of metals onto graphene oxide (GO) is highly important for building suitable contacts when GO or reduced GO (rGO) is used in electronic and electrochemical devices. Moreover, the metal/graphene oxide (M/GO) interface itself sometimes acts as a catalytic site for chemical reactions^1^,^2^. The content of oxygenated functional groups at this interface is expected to be strongly affected by the attachment of metals^3^. Thus, understanding M/GO interfaces at atomic and/or electronic scale may bring about the development of new electronic/spintronic, photochemical, and electrochemical devices because their properties are affected by defects and the content of oxygenated functional groups^4^,^5^,^6^.

The electronic and magnetic properties of transition metals deposited on graphene have been theoretically studied on the basis of *ab initio* density functional theory^7^,^8^,^9^,^10^,^11^,^12^. Au is well-known as a suitable contact material in electronic devices, and has been widely (especially theoretically) studied at Au/graphene or Au/graphite interfaces toward the development of new electronic devices^13^,^14^,^15^,^16^,^17^,^18^. It has been reported that Au atoms attached to graphene nanoribbons move along the edges together with Au–C bonds at high temperature^16^. On the other hand, metal sputtering onto graphene and GO surfaces damages and/or removes the top layer and at the same time changes the GO composition (through reduction)^3^. The compositional changes depend on the metal type^3^, suggesting that some reactions occur between the metal and the GO surface.

Here, the unique phenomenon of metal permeation into GO paper bulk (multi-layered GO film) from an M/GO interface is reported. This permeation occurs at room temperature, especially under humid conditions, and two predominant mechanisms of metal ion/atom permeation are suggested.

## Results

**Figure 1:**
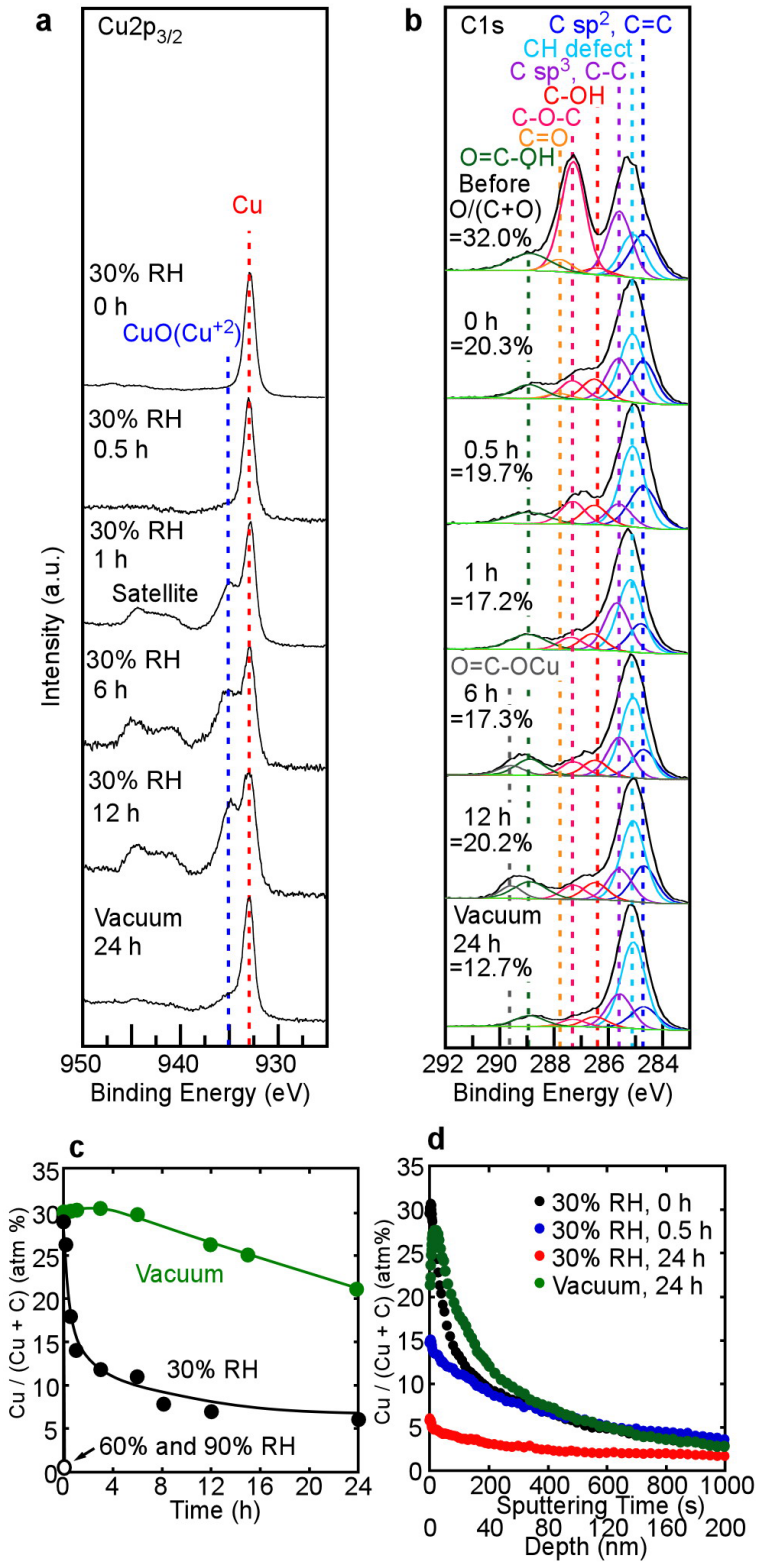
Time dependences of XPS spectra, surface content of Cu, and depth profiles of Cu in the Cu(17 nm)/GO sample. (a) Time dependences of XPS spectra of Cu2p_3/2_ at the Cu/GO interface under 30% RH and vacuum. Metallic Cu changed readily to Cu^2+^ (CuO) under 30% RH but hardly did so under vacuum. (b) Time dependences of XPS spectra of C1s at the Cu/GO interface under 30% RH and vacuum. O in O/(C + O) corresponds to the total amount of O in the oxygenated functions of GO. Weak reduction of GO and formation of Cu(COO)_2_ proceed, but oxidation begins after 12 h. (c) Cu content at the Cu/GO interface as a function of time under humid conditions and vacuum. The Cu content drastically decreased under 60% and 90% RH. (d) Depth profiles of the Cu content at the Cu/GO interface at various times under 30% RH and vacuum. Cu has clearly permeated into the GO bulk.

**Figure 2:**
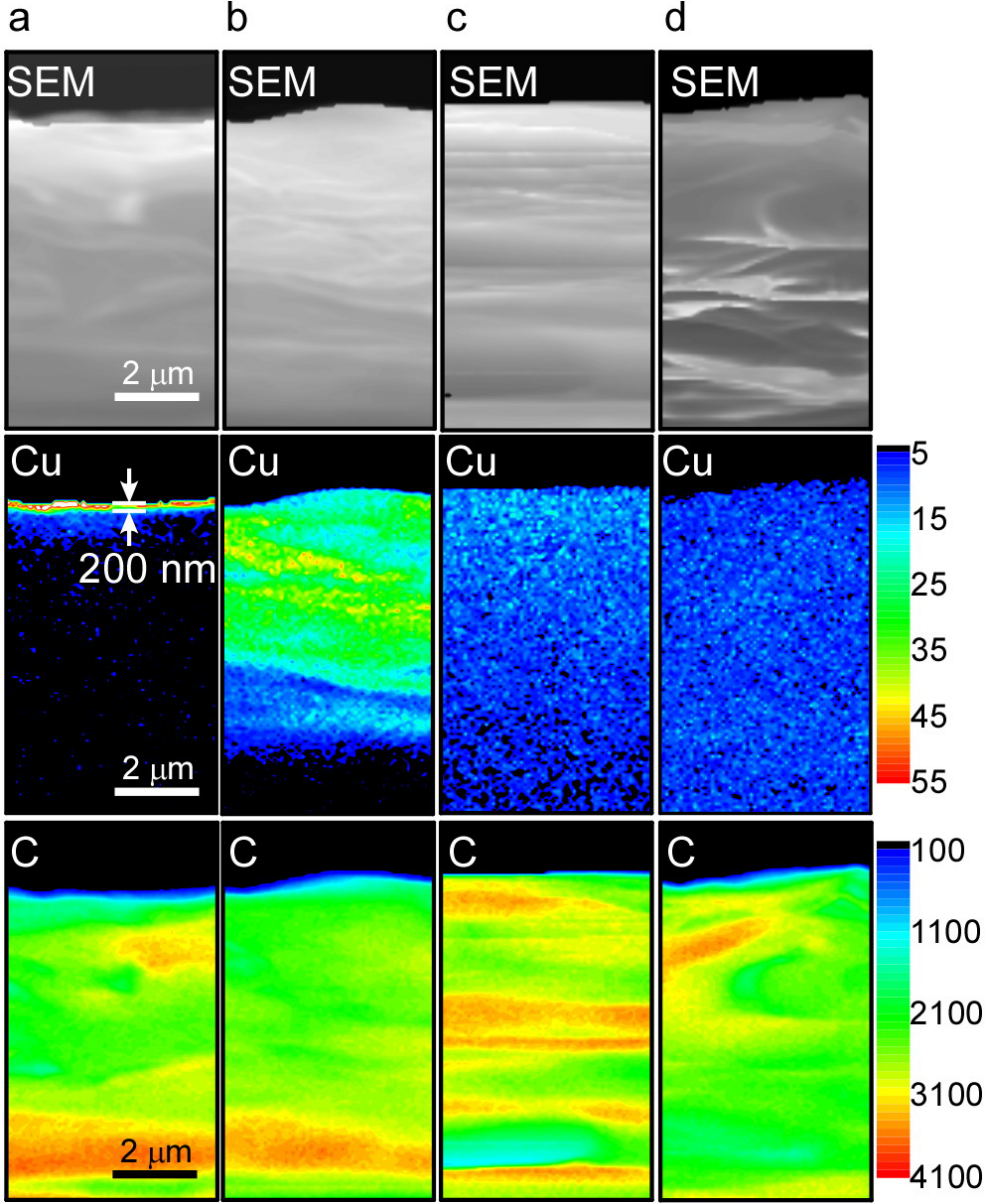
Cross-sectional SEM images and EPMA mappings of Cu and C. (a) As-deposited sample, sample kept at (b) 30% RH for 1 h, (c) 90% RH for 10 min, and (d) 90% RH for 1 h.Scale bars denote the concentrations of Cu and C.

**Figure 3:**
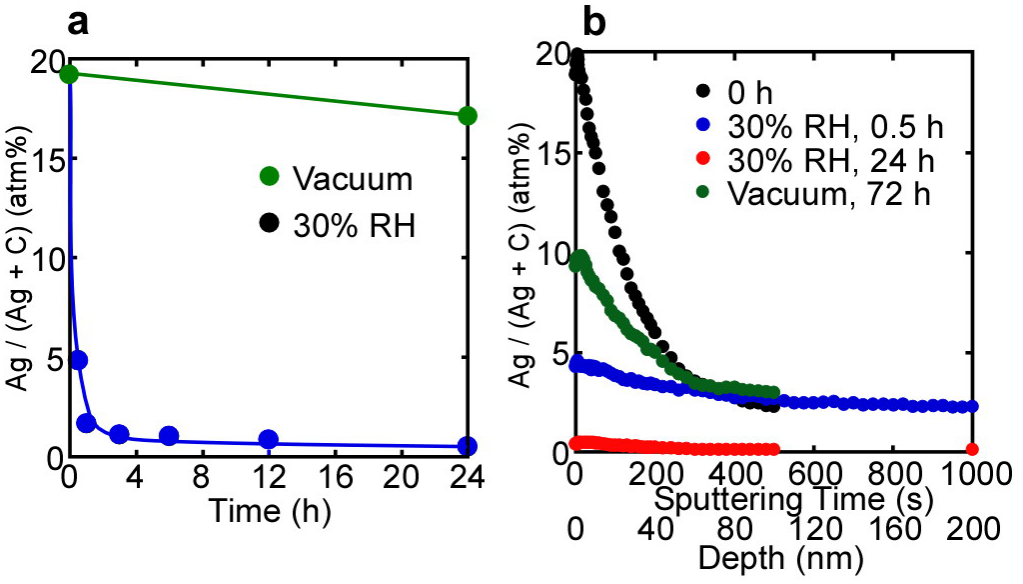
Surface content and depth profiles of Ag in the Ag(17 nm)/GO sample. (a) Ag content at the Ag/GO interface as a function of time under 30% RH and vacuum. The Ag content markedly decreased with time under 30% RH, but barely decreased under vacuum. (b) Depth profiles of the Ag content at the Ag/GO interface at various times under 30% RH and vacuum. Ag has clearly permeated the GO bulk.

**Figure 4:**
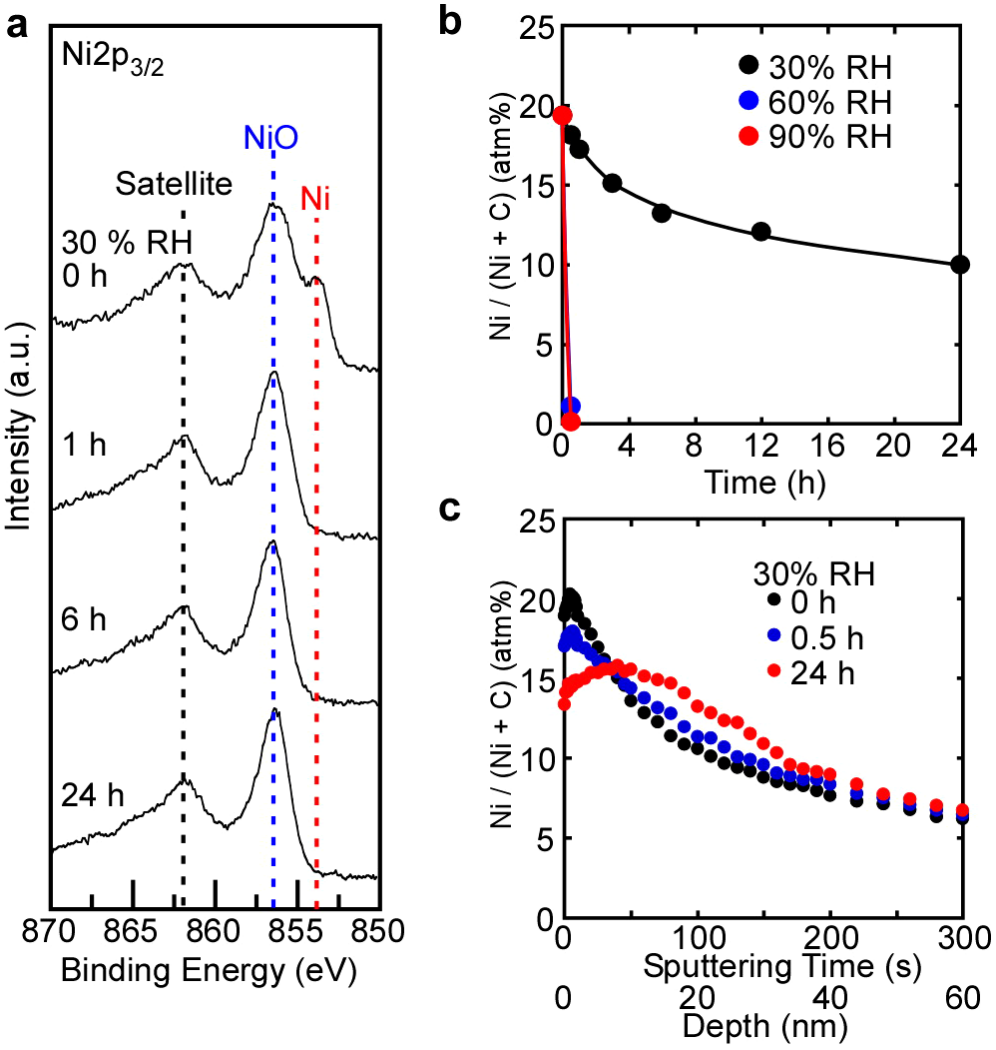
Time dependences of XPS spectra, surface content of Ni, and depth profiles of Ni in the Ni(17 nm)/GO sample. (a) Time dependences of XPS spectra of Ni2p_3/2_ at the Ni/GO interface under 30% RH. Ni metal changed to Ni^2+^ (NiO) under 30% RH immediately after sputtering. (b) Ni content at the Ni/GO interface as a function of time under humid conditions. Ni content drastically decreased with time under 60% and 90% RH, compared with that under 30% RH. (c) Depth profiles of the Ni content at the Ni/GO interface at various times under 30% RH. After 30 min, Ni permeated the GO bulk.

**Figure 5:**
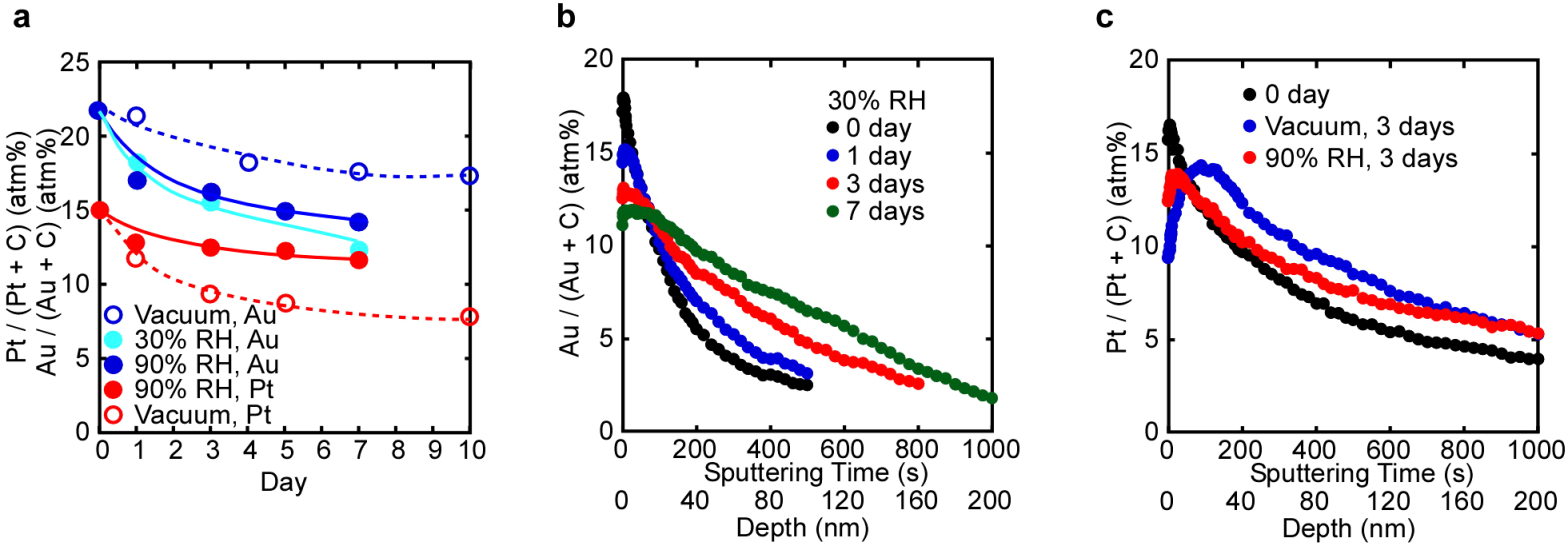
Surface content and depth profiles of Au and Pt in the Au(17 nm)/GO and Pt(17 nm)/GO samples, respectively. (a) Au and Pt content at the Au/GO and Pt/GO interfaces, respectively, as a function of time under humid conditions and vacuum. The Au content decreased with time under humid conditions, but barely did so under vacuum; in contrast, the Pt content decreased under vacuum but barely did so under 90% RH. (b) Depth profiles of the Au content at the Au/GO interface at various times under 30% RH. Au permeated the GO paper from the surface to around 200 nm into the GO bulk after 7 days. (c) Depth profiles of the Pt content at the Pt/GO interface at various times under 90% RH and vacuum. Pt permeated into the GO bulk after several days under vacuum.

After sputtering of metal atoms onto GO, the GO surface was somewhat reduced due to the high energy of the sputtered metal atoms^3^. The X-ray photoelectron spectroscopy (XPS) spectrum^19^ and the cross-sectional field emission scanning electron microscopy (FE-SEM) image of the GO paper without metal deposition are shown in Fig. S1. Although sputtered metal atoms mainly reduced the epoxy groups of the GO paper, the amount of carbonyl groups hardly changed. Cross-sectional SEM images of the GO paper showed that its thickness was about 20 μm, and GO sheets were piled up in parallel without cracks. Simultaneously, the GO surface composition changed depending on time and the type of metal used (Figs. 1,2,3,4,5). The remarkable phenomenon observed here is that permeation of metals occurred from M/GO interface into the GO paper bulk at room temperature at a rate depending on humidity.

Figure 1 shows the time dependences of XPS spectra for (a) Cu2p_3/2_ and (b) C1s binding energies and (c) the Cu content at the Cu(17 nm)/GO interface under humid conditions and vacuum at 25°C (in Fig. 1 (b), the XPS spectrum of GO before sputtering is also shown). Note that Cu^2+^ was produced from Cu metal under humid conditions, and that the Cu content at the interface decreased from around 30% to 6% at <30% relative humidity (RH) and to 0% at ≥60% RH (after 24 h, Fig. 1 (c)). These results imply that Cu changed to Cu^2+^ at the Cu/GO interface (or the GO surface) and then immediately moved into the GO paper bulk under humid conditions. The permeation rate was rather high under high humidity (60% and 90% RH), but low under low humidity. Thus, the presence of vapor is important for fast permeation. According to the XPS analysis of Cu2p_3/2_, Cu^2+^ production was limited under vacuum (Figs. 1 (a) and S2 (a)).

Figure 1 (d) shows depth profiles of the Cu content in the region from the Cu/GO interface to the GO paper bulk under 30% RH and vacuum. In all depth profiles presented here, the GO surface contains a certain amount of Cu because a signal corresponding to Cu is observed at a certain depth even at 0 h. Therefore, the distribution of Cu in depth after a certain time should be evaluated based on comparison with that at 0 h. The decrease of Cu content with time at the surface region (<around 80 nm) suggests fast Cu^2+^ permeation into the bulk. The permeation rate is estimated to be >7 nm/min under 30% RH (>200 nm/30 min, as shown in Fig. 1 (d)). The low content (<5%) of Cu in the region from the surface to a depth of 200 nm in the present Cu/GO sample after 24 h suggested that all of the coated Cu had permeated deeply into the GO bulk. Furthermore, according to the depth profiles (Fig. 1 (d)) and the decrease in Cu surface content from around 30% to 21.5% under vacuum (Fig. 1 (c)), it is possible that Cu also moves as atoms into the GO bulk, although the permeation rate is rather low. The mechanism of the atom diffusion will be similar to those of Au and Pt as described in the later section.

Figure S3 shows XPS spectra of Cu2p_3/2_ and C1s of copper(II) oxalate hemihydrate (Cu(COO)_2_·0.5H_2_O). According to the C1s spectrum of Cu(COO)_2_, the peak originating in the carboxyl group of Cu(COO)_2_ appears near 299.8 eV. Thus, based on Fig. 1 (b) and supplementary Fig. S2 (a) and (b), the formation of Cu(COO)_2_ was clear when the peak appeared at 299.8 eV. In particular, under 90% RH, the oxidation of Cu to Cu^2+^ (*i.e.*, production of Cu(COO)_2_) was remarkably high, as shown in Fig. S2. Then, the XPS peak intensity decreased with time in the case of 90% RH. These results indicate that Cu^2+^ in Cu(COO)_2_ produced at the surface permeates the GO paper bulk at the same time H^+^ exchange occurs (mainly at COOH groups), especially under high humidity. The permeation rate visibly decreased with increasing the film thickness of Cu, judging from the comparison of the surface content changes in Fig. 1 (c) (Cu film thickness: 17 nm) and Fig. S2 (c) (Cu film thickness: 400 nm). No decrease in Cu surface content was observed under vacuum for the thick Cu film (400 nm), as seen in Fig. S2 (c). These results do not mean that Cu^2+^ ion and Cu atom scarcely diffuse for the case of thick films, because the compositions hardly changes for a large amount of Cu of thick film.

Figure 2 shows a mapping of Cu permeating into GO paper, as observed by cross-sectional electron probe microanalysis (EPMA). From cross-sectional EPMA of the as-deposited sample (Fig. 2 (a)), the thickness of deposited Cu on GO paper was estimated to be about 200 nm. Although this is about 10-fold the thickness of the Cu layer on the sample estimated by XPS depth profile analysis, it is in fact plausible in light of the fact that the sputtering time was 10 times longer for that sample. Cross-sectional SEM images and XRD patterns of GO papers revealed closely packed self-assembled GO sheets. These results suggest that the structure of the GO paper was seldom affected by metal permeation. Cu permeation was clearly observed under humid conditions at 25°C (Fig. 2 (b)–(d)). The distribution of Cu extended from the M/GO interface to a depth of about 6 μm under 30% RH for 1 h (Fig. 2 (b)). However, when the Cu-deposited sample was kept under 90% RH, in only 10 min, the Cu permeated the GO paper to a remarkable depth extending to the bulk of the GO paper (Fig. 2 (c)). For the EPMA image of the sample kept under 90% RH for 1 h, the Cu concentration in the GO paper was nearly uniform (Fig. 2 (d)). These EPMA results suggest that Cu permeation into the GO paper took place rather quickly under high humidity.

The oxidation of Cu led to the reduction of GO under humid conditions, as shown in Fig. 1 (b), where after 12 h GO began to oxidize due to the termination of Cu^2+^ production. Based on the above results, the following reactions at the surface took place under humid conditions if CH was produced. As a matter of course, other reduction reactions with respect to GO proceed simultaneously. 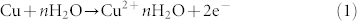

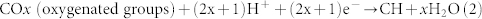


Some of the hydrated Cu^2+^ are immediately exchanged with H^+^, mainly at COOH sites, to form Cu(COO)_2_ at the surface, and then move into the interlayers of the GO paper aided by water molecules and H^+^ exchange. The permeation model is illustrated in the discussion section.

Figure 3 shows (a) the time dependences of the Ag content at the Ag(17 nm)/GO interface and (b) the depth profiles covering the region from the interface to the bulk. Ag permeation occurred much faster under humid conditions than under vacuum, similar to Cu. Although it was difficult to distinguish between Ag and Ag^+^ from the binding energy of Ag3d (supplementary Fig. S4), Ag_2_O (that is, Ag^+^ ion) formation was observed from the XPS spectra of O1s (Fig. S4). In particular, its formation under vacuum was clear because Ag^+^ in Ag_2_O hardly moved from the GO surface into the bulk. The O atom in Ag_2_O seems to be taken from C−O−C groups because the C−O−C content correspondingly decreased with the formation of Ag_2_O, especially under vacuum (Fig. S4). In any case, the Ag^+^/Ag redox reaction, similar to reaction (1) together with reaction (2), occurs at the Ag/GO interface. Consequently, Ag changes to Ag^+^ at the interface and then moves into the bulk through the interlayers of the GO paper with the aid of water molecules. The permeation sites are similar to those of H^+^, which correspond to bonding sites between C−O−C (and/or OH) groups and water molecules^5^,^20^,^21^. According to cross-sectional EPMA mappings of Ag (Fig. S5), Ag permeated the GO paper at an extremely high rate under 30% RH.

Figure 4 shows XPS spectra of (a) Ni2p_3/2_, (b) the time dependences of Ni content at the Ni/GO interface, and (c) depth profiles covering the region from the surface to the bulk. Ni permeation occurred from the Ni/GO interface into the GO bulk, as shown in Fig. 4 (b) and (c), where the permeation rate increased remarkably under 60% and 90% RH. Oxidation of Ni metal to Ni^2+^ occurred at the surface immediately after sputtering, and then Ni metal disappeared with time following a redox reaction similar to those of Cu and Ag. In fact, GO reduction proceeded with time, as shown in supplementary Fig. S6. Figure S7 shows cross-sectional SEM images and EPMA mappings of Ni and C for the Ni-deposited sample. The layer of Ni remained at the interface under 30% RH for 1 h, which suggests that the permeation rate of Ni into the GO paper was low compared with Cu and Ag. In the case of 90% RH, Ni permeated the GO paper to a sufficient depth, similarly to Cu. These EPMA results were in close agreement with the XPS depth profile data.

Figure 5 shows (a) the time dependences of Au and Pt content at the Au/GO and Pt/GO interfaces, respectively, and depth profiles for (b) Au and (c) Pt. Au and Pt were always in the form of elemental metals, as clear from supplementary Fig. S8. Au permeation occurred, although the rate was rather low compared to that of the metal ions examined above, as already stated. The permeation rate for Au atoms increased slightly with increasing the humidity. On the other hand, Pt permeation occurred with relative ease under vacuum, but with difficulty under humid conditions. The depth profiles of Au and Pt are different from each other in terms of metal distribution. Au was smoothly distributed in depth, while the Pt distribution reached maximum at around 30 nm after 3 days. Figure S9 shows cross-sectional SEM images and EPMA mappings of Au and C. In the EPMA images of the sample kept for 10 days under 30% RH, slight permeation of Au into the GO paper bulk was observed. However, EPMA could not provide clear evidence of Pt permeation into the GO paper because of the low spatial resolution. According to the XPS depth profile, Au permeated GO from the surface to a depth of around 200 nm, whereas the concentration of Pt in the region from the surface to around 200 nm hardly changed during the experiment. This further confirms that Au and Pt permeate the GO paper bulk as metal atoms at room temperature.

## Discussion

The difference in distribution between Pt and Au may be explained by the M–C bond energy^22^,^23^,^24^, which is higher for Pt–C than for Au–C, leading to relatively smooth permeation for Au. In this regard, the in-plane Au and Pt atom permeation on substrates such as GO and highly oriented pyrolytic graphite (HOPG) at room temperature has been reported^25^,^26^,^27^. Au atom permeation into Si bulk has also been reported to occur even at room temperature^28^ with a permeation rate of around 11 nm/10 days (Fig. 2 in ref. 28). In contrast, the present permeation rate of Au into GO bulk was about 110 nm/7 days (Fig. 5 (b). This value was estimated from the difference in the sputtering depth at which the Au/(C + Au) = 0.05). Although it is impossible to determine the penetration rate accurately from our EPMA results at the resolution used in the study, Au permeation occurred at a rate of 200 nm/10 days when estimated from the penetration depth of metal, as determined by EPMA (Fig. S9). These permeation rates of Au into GO bulk are about 10 times faster than in case of Au permeation into Si. Some theoretical studies suggest that defects or edges in graphene act as permeation sites for metal atoms^14^,^15^,^16^,^23^,^24^. GO has many defects and edges, both with and without oxygenated functions, which likely act as permeation sites for Au and Pt atoms and result in the permeation of Au and Pt atoms into the GO bulk even at room temperature in the present cases. The effect of water molecules in this phenomenon suggests an important relation of oxygenated functions to the atom permeation mechanism because water molecules interact with these functions, especially in the interlayers.

**Figure 6:**
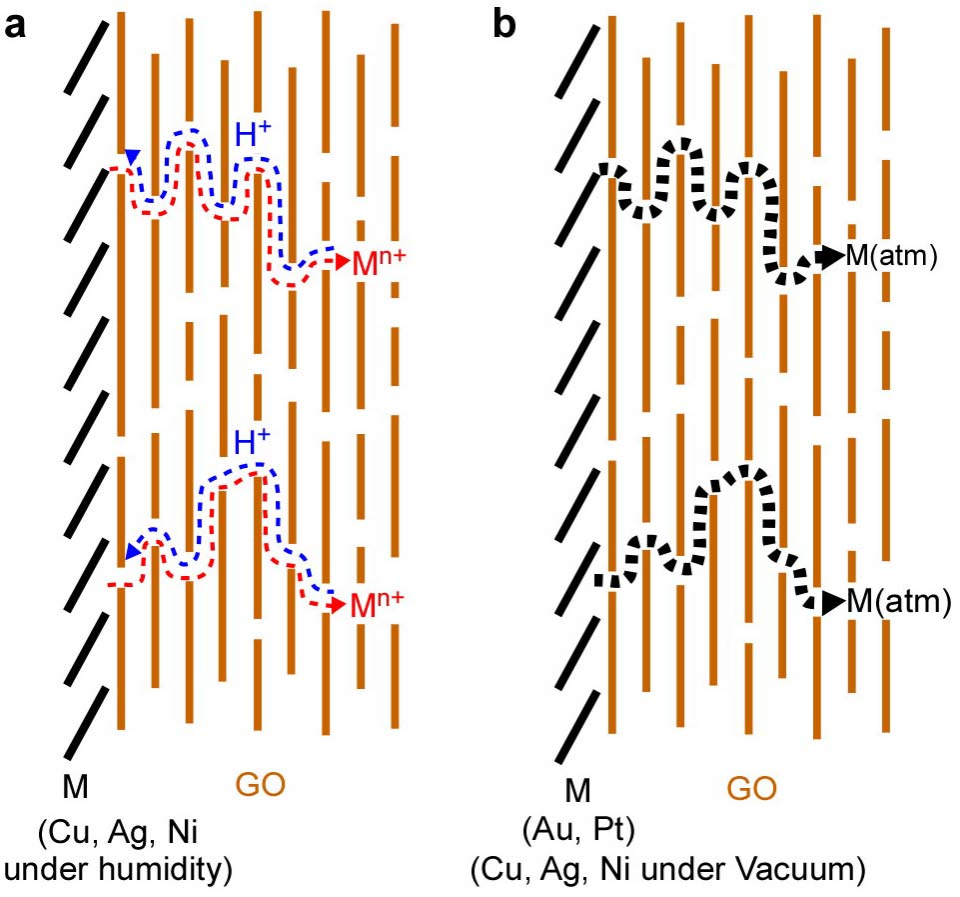
Permeation models for Cu, Ag, Ni, Au, and Pt metals. (a) Permeation model for Cu, Ag, and Ni. Metals are oxidized to the corresponding ions at the surface and then permeate into the GO bulk with simultaneous proton exchange. (b) Permeation model for Au and Pt, which permeate the GO bulk as atoms via defects and/or edges. Other metal (Cu, Ni, Ag) will diffuse as atoms with low diffusion rate.

Two models of the metal permeation mechanism in the M/GO samples are illustrated in Fig. 6. The first model involves metal ion permeation together with H^+^ exchange in the GO interlayers (Fig. 6 (a)). In this case, the permeation rate is rather high under humid conditions, even at room temperature. This mechanism holds for Cu^2+^, Ni^2+^, and Ag^+^ permeation in the Cu/GO, Ni/GO, and Ag/GO samples, respectively. In this regard, ion transport through the GO interlayers has been observed for some other ions as well, and the permeation mechanism is similar to that in the present cases^29^,^30^.

The second mechanism accounts for Au and Pt atom permeation in the Au/GO and Pt/GO samples (Fig. 6 (b)). It should be noted that Au and Pt atoms move in the GO paper even at room temperature, although the permeation rate is low compared to the cases of metal ions. In these cases, the permeation sites are defects and/or edges, which are abundant in the interlayers of GO paper. Similar atom diffusion may be occur for Cu, Ag and Ni, because small diffusions were observed in vacuum condition (Figs. 1(a) and 3(a)). As shown in Fig. S10, metal deposited on HOPG, which has neither defects nor edges on the surface, hardly permeated into the bulk. In the case of Au, AuS formation under vacuum was observed in S2p XPS spectra^31^. Figure S11 shows time dependences of XPS spectra for S2p under (a) vacuum and (b) 90% RH and (c) S content at the Au/GO interface under vacuum and 90% RH. The amount of AuS increased with increasing time until 7days under vacuum. The formation of AuS was suggested by trace amounts of SO_4_^2−^ present in the GO paper prepared by the Hummers' method (S/C = 0.005). If AuS were generated at the GO surface, internal permeation of Au would be suppressed and the atomization of Au would be inhibited. However, the concentration of S at the surface was almost constant under humid conditions (Fig. S11(c)). AuS hardly form under humid conditions (Fig, S11(b)) because the SO_4_^2−^ ions adsorbed on Au were hydrated. Hydrate formation likely promoted the permeation of Au under humid conditions. In the case of Pt, permeation was faster at the GO surface under vacuum than under humid conditions. Chusuei *et al.* have reported that COO(Pt) or C( = O)COPt forms when Pt nanoparticles are deposited on chemically oxidized carbon nanotubes^32^. Pt atoms will permeate until adsorbing to the carboxyl groups inside GO. Under humid conditions, adsorption of protons to the carboxyl groups that serve as sites for metals to move would inhibit the permeation of Pt. The XPS depth profiles shown in Fig. 5(c) also suggest that Pt permeation took place near the GO surface only. As a result, the amount of Pt decreased at the GO surface. Figure S12 shows C1s XPS spectra for specimens stored under (a) vacuum and (b) 90% RH for 10 days. When Pt-deposited GO was stored for 10 days under 90% RH, the amount of oxygenated functional groups decreased. This result means that Pt catalyzed reduction of GO under humid conditions^33^. Since the number of sites for Pt permeation decreased when GO was reduced, Pt permeation was suppressed under humid conditions. The details of the permeation mechanisms for Au and Pt are under investigation for reduced GO paper and GO paper with various added anions.

Furthermore, in order to evaluate the implantation effect caused by impinging Ar^+^ ions during XPS depth profiling, permeation of metals such as Cu and Pt in vacuum was investigated after applying Ar^+^ ion sputtering for 30 s to the metal-deposited samples (Fig. S13). Although the surface concentration of metals decreased immediately after Ar^+^ ion sputtering, there was almost no further decrease in concentration in the next 24 h. The influence of Ar^+^ ion impingement for 30 s was hardly noticeable in the shape of the XPS depth profiles of the samples kept in vacuum for 24 h.

In conclusion, metal permeation at M/GO interfaces was observed at 25°C, where the metals were deposited onto the surface of GO paper by sputtering. In the cases of M = {Cu, Ag, Ni}, the metals were oxidized to the corresponding ions (Cu^2+^, Ag^+^, and Ni^2+^) by redox reactions involving the reduction of GO immediately after metal deposition. These ions then quickly permeated through the interlayers into the GO paper bulk. These reactions and fast permeation occurred with ease under humid conditions but with difficulty under vacuum. Au and Pt atoms also permeated the GO paper bulk, although the permeation rates were low. In these cases, abundant defects and/or edges in the GO paper acted as permeation sites. Other metals are also likely to be able to permeate GO paper bulk as atoms. Consequently, GO paper interlayers have unique properties allowing water, metal ions and other atoms to permeate them while providing a shield against gas molecules^34^. These findings are expected to provide important information about GO used in various (especially electrochemical) devices, where ionic movement potentially affects the functionalities.

## Methods

Graphite oxide was prepared by the well-known Hummers' method^35^ using 98% graphite powder (Wako Pure Chemical Industries, Ltd.) as the starting material. The GO nanosheet solution (3 g/L) was prepared by exfoliation of GO in pure water by ultrasonication and centrifuged at 10000 rpm to remove any aggregated GO. The GO nanosheet solution was filtered using a membrane filter having a 0.4 μm pore size. The GO paper was obtained by exfoliation from the filter, followed by drying at room temperature under vacuum. The thickness of the GO paper was about 20 μm according to cross-sectional FE-SEM performed using a SU-8000 field-emission SEM (Hitachi High Technologies, Ltd.). Metals (Cu, Ag, Ni, Au, and Pt, The Nilaco Co.) were deposited onto the GO paper surface using a K575X sputter coater (Emitech Ltd.) to a thickness of about 17 nm for acquiring XPS depth profiles and about 200 nm for performing cross-sectional EPMA, unless otherwise stated. The sputtering time of the samples used for cross-sectional EPMA was set to 10 times that for the samples used for XPS depth profile acquisition. After keeping the M/GO samples under vacuum and under humid conditions at 25°C, the following XPS analyses were performed. XPS was carried out using a Sigma Probe XPS analysis system (Thermo Fisher Scientific Inc.), with monochromatic Al–Kα radiation in order to analyze the binding energies and quantitatively analyze the elements in the M/GO samples. The instrument work function was calibrated to give a binding energy of 83.95 eV for an Au4f_7/2_ line for metallic gold, and the spectrometer dispersion was adjusted to give a binding energy of 367.85 eV for metallic Ag3d_5/2_ and 932.65 eV for metallic Cu2p_3/2_. The instrument base pressure was 1 × 10^−9^ mbar. The depth profiles were obtained by sputtering 3 eV Ar^+^ ions, during which the background pressure in the chamber was 1.0 × 10^−7^ mbar and a current density of 170 μA cm^−2^ was maintained. Under these conditions, the sputtering rate was approximately 0.2 nm s^−1^ for metal-deposited GO films^36^,^37^,^38^. The sputtering rate was set to be substantially lower than the commonly used rate in order to minimize the influence of metal permeation induced by Ar^+^ ion impingement. The reference samples for XPS measurement were copper oxalate hemihydrate (Cu(COO)_2_·0.5H_2_O, Wako Pure Chemical Industries, Ltd.) and highly oriented pyrolytic graphite (HOPG, NT-MDT Co., Type-ZYB). The cross-sections of the samples were analyzed by means of EPMA (EPMA-1720H, Shimadzu Co., Ltd.) with 5 wavelength-dispersive spectrometers. The experiments were conducted with a focused electron beam current of 5 nA and an acceleration voltage of 15 kV. The samples subjected to cross-sectional EPMA measurements were pulverized in liquid nitrogen and were immediately introduced into the vacuum chamber.

## Author Contributions

C.O., M.K. and Y.M. designed the experiments and analyzed the data. Y.M. and M.K. mainly wrote the manuscript. C.O. and K.H. was prepared GO and M/GO. M.K. performed XPS measurement and analysis. K.H. performed EPMA measurement. H.T., M.R.A., T.T. and A.F. made the plane and advised on the optimization of the experiments. All authors discussed and commented on the manuscript.

## Supplementary Material

Supplementary Information Supplementary Data
