# Supplementary material for: Metal Permeation into Multi-layered Graphene Oxide
Source: Sci Rep. 2014 Jan 13;4:3647. doi: 10.1038/srep03647 (PMC3888985; doi:10.1038/srep03647)
Supplement: Supplementary Information — Supplementary Data [file srep03647-s1.doc]

*Supplementary Information*

Metal Permeation into Multi-layered Graphene Oxide

Chikako Ogata, Michio Koinuma*, Kazuto Hatakeyama, Hikaru Tateishi, Mohammad Razaul Asrori, Takaaki Taniguchi, Asami Funatsu, Yasumichi Matsumoto*

Figure S1. (a) XPS spectrum of C1s of the GO paper without metal deposition. (b) Cross-sectional FE-SEM image of GO paper.

Figure S2. Time dependences of XPS spectra and surface content of Cu in the Cu(400 nm)/GO samples under 90% RH and vacuum. (a) Time dependences of XPS spectra of Cu2p_3/2_ at the Cu/GO interface. Metallic Cu changed to Cu^2+^ (CuO) immediately after sputtering under 90% RH but not under vacuum. (b) Time dependences of XPS spectra of C1s at the Cu/GO interface. Cu(COO)_2_ was produced immediately after sputtering, but its content decreased with time. (c) Cu content at the Cu/GO interface as a function of time under 90% RH and vacuum. The Cu content decreased under 90% RH but not under vacuum.

Figure S3 | XPS spectra of copper(II) oxalate (Cu(COO)_2_). (a) Cu2p_3/2_ and (b) C1s

Figure S4. Time dependences of XPS spectra of Ag3d, C1s, and O1s at the Ag(17 nm)/GO interface under 30% RH and vacuum. Epoxide groups (C1s) remained even after 24 h under 30% RH. According to the O1s spectra, Ag_2_O (Ag^+^) was formed, and its peak intensity decreased with time because of Ag^+^ permeation into the GO bulk under 30% RH. In contrast, this peak intensity was retained even after 24 h under vacuum because permeation into the GO bulk barely occurred under vacuum.

Figure S5. Cross-sectional SEM images and EPMA mappings of Ag and C. (a) As-deposited sample and (b) sample kept at 30% RH for 1 h. Scale bars denote the concentrations of Ag and C.

Figure S6. Time dependences of XPS spectra of C1s at the Ni(17 nm)/GO interface under 30% RH. Oxygen content decreased with time.

Figure S7. Cross-sectional SEM images and EPMA mappings of Ni and C. (a) As-deposited sample, sample kept at (b) 30% RH for 1 h, (c) 90% RH for 10 min, and (d) 90% RH for 1 h. Scale bars denote the concentrations of Ni and C.

Figure S8. Time dependences of XPS spectra of Au4f, Pt4f, and C1s at the Au/GO (under 30% RH) and Pt/GO (under 90% RH) interfaces. (a) XPS spectra of Au4f and C1s at the Au/GO interface. (b) XPS spectra of Pt4f and C1s at the Pt/GO interface. Both Au and Pt were present in metal form, and the composition of the oxygenated groups barely changed with time.


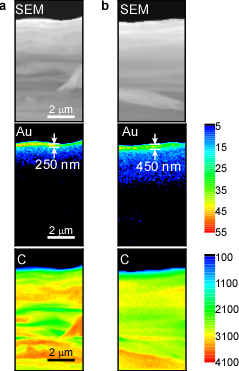


Figure S9. Cross-sectional SEM images and EPMA mappings of Au and C. (a) As-deposited sample and (b) sample kept in vacuum for 10 days. Scale bars denote the concentration of Au and C.

Figure S10. (a) Depth profiles of the Pt content at the Pt/HOPG interface after 7 days under vacuum. (b) Depth profiles of the Cu content at the Cu/HOPG interface after 1 h under 90% RH.


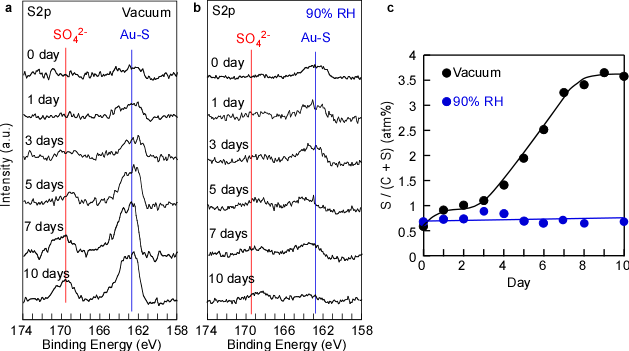


Figure S11. Time dependences of XPS spectra and surface content of S. Time dependences of S2p XPS spectra at the Au/GO interface under (a) vacuum and (b) 90% RH. (c) S content at the Au/GO interface as a function of time under vacuum and 90% RH.


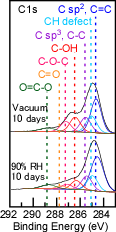


Figure S12. C1s XPS spectra at the Pt(17 nm)/GO interface under 90% RH and under vacuum for 10 days.

Figure S13. (a) Cu and Pt content at the Cu/GO and Pt/GO interfaces, respectively, as a function of time under vacuum. (b) Depth profiles of the Cu and Pt content at the Cu/GO and Pt/GO interfaces, respectively, after 1 day under vacuum.
